# Structural and functional connectivity in the default mode network in 22q11.2 deletion syndrome

**DOI:** 10.1186/s11689-015-9120-y

**Published:** 2015-08-01

**Authors:** Maria Carmela Padula, Marie Schaer, Elisa Scariati, Maude Schneider, Dimitri Van De Ville, Martin Debbané, Stephan Eliez

**Affiliations:** Office Médico-Pédagogique, Department of Psychiatry, University of Geneva, Rue David-Dufour 1, Case Postale 50, 1211 Genève 8, Switzerland; Stanford Cognitive and Systems Neuroscience Laboratory, Stanford University, Stanford, CA USA; Department of Radiology and Medical Informatics, University of Geneva, Geneva, Switzerland; Medical Image Processing Lab, Institute of Bioengineering, Ecole Polytechnique Fédérale de Lausanne, Lausanne, Switzerland; Adolescence Clinical Psychology Research Unit, Faculty of Psychology and Educational Sciences, Geneva, Switzerland; Research Department of Clinical, Educational and Health Psychology, University College London, London, U K; Department of Genetic Medicine and Development, University of Geneva, Geneva, Switzerland

**Keywords:** Resting-state fMRI, DTI, Tractography, Maturation, Positive symptoms, Schizophrenia

## Abstract

**Background:**

The neural endophenotype associated with 22q11.2 deletion syndrome (22q11DS) includes deviant cortical development and alterations in brain connectivity. Resting-state functional magnetic resonance imaging (fMRI) findings also reported disconnectivity within the default mode network (DMN). In this study, we explored the relationship between functional and structural DMN connectivity and their changes with age in patients with 22q11DS in comparison to control participants. Given previous evidence of an association between DMN disconnectivity and the manifestation of psychotic symptoms, we further investigated this relationship in our group of patients with 22q11DS.

**Methods:**

T1-weighted, diffusion, and resting-state fMRI scans were acquired from 41 patients with 22q11DS and 43 control participants aged 6 to 28 years. A data-driven approach based on independent component analysis (ICA) was used to identify the DMN and to define regions of interest for the structural and functional connectivity analysis. Prodromal psychotic symptoms were assessed in adolescents and adults using the positive symptom scores of the Structured Interview of Prodromal Syndromes (SIPS). Connectivity measures were compared between groups and correlated with age. Repeating the between-group analysis in three different age bins further assessed the presence of age-related alterations in DMN connectivity. Structural and functional connectivity measures were then correlated with the SIPS scores.

**Results:**

A simultaneous reduction of functional and structural connectivity between core medial nodes of the DMN was observed. Furthermore, structural connectivity measures significantly increased with age in the control group but not in patients with 22q11DS, suggesting the presence of an age-related alteration of the DMN structural connections. No correlations were found between the DMN disconnectivity and expression of prodromal symptoms in 22q11DS.

**Conclusions:**

These findings indicate the presence of functional and structural DMN disconnectivity in 22q11DS and that patients with 22q11DS fail to develop normal structural connections between medial DMN nodes. This suggests the presence of altered neurodevelopmental trajectories in 22q11DS.

**Electronic supplementary material:**

The online version of this article (doi:10.1186/s11689-015-9120-y) contains supplementary material, which is available to authorized users.

## Background

Chromosome 22q11.2 deletion syndrome (22q11DS) is a neurodevelopmental disorder associated with a broad phenotypic expression including clinical, cognitive, and psychiatric manifestations [1]. Cognitive problems are highly variable and comprise alterations in learning, language, working memory, and executive functions [2–5]. The psychiatric phenotype is characterized by social impairments, anxiety, and the manifestation of schizophrenia-like disorders, which occur in 30 % of adults affected by the syndrome [6, 7].

Cognitive processes result from the dynamic interaction between brain regions that communicate with each other through structural and functional connections [8, 9]. In order to ensure an efficient synchrony between integrated brain areas, these connections evolve during neurodevelopment [10]. For instance, fractional anisotropy (FA) significantly increases during childhood and adolescence signifying an increase of myelination that ensures more rapid signal conduction [10–12]. Functional networks undergo similar processes of integration and specialization [13–15]. These maturation processes are altered in neurodevelopmental and psychiatric disorders [16–18], resulting in the onset of cognitive, behavioral, and psychological impairments.

Structural neuroimaging studies based on diffusion tensor imaging (DTI) conducted in patients with 22q11DS reported altered white matter microstructure [19–30]. Also, a previous study by our group using tractography reported impairments in limbic, parietal, and fronto-temporal connections [31]. Specifically, reduced fronto-temporal connectivity was considered a vulnerability factor for the development of schizophrenia in 22q11DS [31]. Moreover, studies based on resting-state fMRI (rs-fMRI) reported functional connectivity alterations within the default mode network (DMN) in 22q11DS [32, 33].

The DMN is one of the most described resting-state networks (RSNs), active in the absence of cognitive tasks and involved in self-referential functions [34, 35]. The DMN includes a collection of spatially distinct regions covering parts of the anterior and the posterior medial areas of the brain as well as the lateral parietal cortices. Anteriorly, the DMN includes the medial prefrontal (mPFC) and the anterior cingulate cortices (ACCs); lateral DMN components have been identified in the bilateral inferior parietal lobule (IPL) and in the medial temporal lobes (MTLs); posteriorly, it comprises the posterior cingulate cortex (PCC) and the precuneus [36, 37].

The first studies investigating DMN connections adopted one imaging modality, either fMRI [38–45] or DTI [43, 46, 47]. More recently, studies began to combine the two modalities in healthy participants [48–52], reporting significant correlations between the DMN structural and functional connections. Similar studies have also been conducted in clinical samples of schizophrenic patients, reporting, for instance, a concomitant impairment in structural and functional DMN connectivity [53] and reduced coherence between structural and functional connections within the DMN [54].

In 22q11DS, a previous study from our group [32] conducted an ICA analysis aiming at investigating the organization of resting-state networks in this population of patients. This study provided evidence for alterations in the functional DMN connectivity, but did not specify which regions of the DMN were disconnected [32]. Indeed, differences between control participants and 22q11DS were evaluated by comparing the spatial activation maps. Subsequent work by Schreiner and colleagues further showed functional disconnections between the anterior and the posterior DMN nodes in 22q11DS [33]. In this context, we further aimed to investigate the within-DMN structural connectivity using an ROI-based approach to assess if the previously reported alterations in the DMN functional connections were associated to alterations in the underlying white matter tracts. The specific interest in studying the DMN structural and functional connectivity in 22q11DS was also motivated by the observation that such patterns of disconnectivity in the DMN have been reported in several neurodevelopmental disorders [44, 45, 47, 55, 56], including schizophrenia [53, 54], suggesting that DMN connectivity could represent a promising endophenotype for neurodevelopmental diseases.

Moreover, previous studies reported the presence of alterations in the trajectories of structural cortical development in individuals with 22q11DS [57–60]. Given this evidence, and considering the normal development of white matter in healthy people [61, 62], we investigated if structural and functional connectivity measures increased with age in our group of control and 22q11DS participants. In addition, we conducted the analysis of functional and structural connectivity in three age subgroups, corresponding to children, adolescents, and young adults.

Finally, we explored whether changes in the DMN connectivity were associated with specific psychiatric symptoms. Evidence suggests that altered connectivity between medial nodes of the DMN is associated with the presence of psychotic symptoms in patients who are affected by, or are at, high risk of schizophrenia [32, 53, 54, 63]. Also, altered DMN connectivity at rest and white matter impairments in the cingulum bundle are associated with prodromal psychotic symptoms in patients with 22q11DS [20, 32]. To further establish if alterations in the DMN connectivity were associated with early symptoms of psychosis in 22q11DS, we investigated the relationship between structural and functional DMN connectivity and the Structured Interview of Prodromal Syndromes (SIPS, [64]) positive scores.

## Methods

### Participants

The group of patients with 22q11DS included 41 participants aged between 8 and 28 years (mean age = 17.1 ± 5.3; 17 males). The IQ of the participants was measured using the Wechsler Intelligence Scale for Children-III [65] or the Wechsler Adult Intelligence Scale-III for adults [66]. The mean IQ was 67 ± 10.6. Sixteen participants (39 %) were under medication at the time of testing. Between them, nine patients were taking methylphenidate, three antipsychotics, three antidepressants, and one antiepileptic. The presence of psychiatric disorders was evaluated during a clinical interview with the participants and their parents using the Diagnostic Interview for Children and Adolescents Revised (DICA-R, [67]) and the psychosis supplement from the Kiddie-Schedule for Affective Disorders and Schizophrenia Present and Lifetime version (K-SADS-PL, [68]) for individuals below 18 years. For adult participants, we used the Structured Clinical Interview for DSM-IV Axis I Disorders (SCID-I, [69]). Twenty-two patients (54 %) met criteria for an axis-I psychiatric disorder at the time of the visit: seven were affected by anxiety disorders, five by attention deficit hyperactivity disorders (ADHDs), three by psychotic disorders, two by mood disorders. The remaining five patients were affected by more than one psychiatric disorder. In addition, we used the Structured Interview for Prodromal Syndromes (SIPS, [64]) to identify the subjects with an ultra high risk (UHR) state. As the administration of the interview requires intact cognitive functioning, six participants were unable to complete this assessment because they were too young (under 12 years old) and two because they had severe cognitive deficits. The mean SIPS scores for each positive (from P1 to P5) and negative (from N1 to N6) subscale are reported in Additional file 1: Table S1. After excluding the three subjects that presented psychotic disorders, five subjects fulfilled the three SIPS criteria for a UHR state (presence of a brief intermittent psychotic symptom prodromal syndrome and/or attenuated positive symptom prodromal syndrome and/or genetic risk and deterioration prodromal syndrome).

Forty-three control participants aged between 6 and 25 years (mean age = 16.1 ± 4.5; 15 males) were recruited among healthy siblings (*N* = 21) of the patients, as well as from the Geneva state school system (*N* = 22). No demographic differences were observed between the two control groups (*p* > 0.42). None of the healthy participants had a past or present history of neurologic or psychiatric disorders, and the mean IQ was 108.4 ± 13.9. Written informed consent was received from all participants or their parents, and the study was approved by the Institutional Review Board of Geneva University School of Medicine.

A total of 55 participants had to be excluded from the original sample (80 patients with 22q11DS, 59 control participants) due to the multimodal nature of the study and the need for good quality data using both the DTI and the resting-state fMRI techniques (Table 1).

**Table 1:** Subjects’ exclusion

No. subjects excluded	Reason for exclusion
2 controls (1 male); 5 22q11DS (2 males)	Moved during the diffusion tensor imaging (DTI) and the resting-state fMRI acquisition
4 controls (2 males); 8 22q11DS (4 males)	Moved during the DTI but not the resting-state fMRI acquisition
4 controls (3 males); 5 22q11DS (1 male)	Moved more than 3 mm in translation or 3° in rotation during the resting-state acquisition
6 controls (4 males); 8 22q11DS (6 males)	The field of view did not include the entire cortex in the DTI or in the functional images
3 22q11DS (males)	Fell asleep during the resting-state acquisition
Tot. 16 controls; 39 22q11DS	

Some of the participants in our final group of subjects were included in our previous studies using either DTI [31] or resting-state acquisitions only [32, 70]. Among the 84 participants, 14 control participants and 12 patients were included in [32], 9 control participants and 5 patients in [31], and 32 controls and 29 patients in [70].

### Image acquisition

A Siemens Trio 3 Tesla scanner was used to acquire anatomical, diffusion-weighted, and functional resting-state imaging data during the same scanning session. The T1-weighted sequence was collected with a 3D volumetric pulse using the following sequence: TR = 2500 ms, TE = 3 ms, flip angle = 8°, acquisition matrix = 256 × 256, field of view = 22 cm, slice thickness = 1.1 mm, and 192 slices. For diffusion tensor imaging (DTI), the following parameters were used: number of directions = 30, *b* = 1000 s/mm^2^, TR = [8300–8800] ms, TE = 82 ms, flip angle = [90–180]°, acquisition matrix = 128 × 128, field of view = 25.6 cm, 64 axial slices, and slice thickness = 2 mm. The rs-fMRI sequence was acquired over 8 min and consisted of 200 blood-oxygenation-level-dependent (BOLD) images (TR = 2400 ms, TE = 30 ms, 38 axial slices, slice thickness = 3.2 mm, flip angle = 85°, acquisition matrix = 94 × 128, field of view = 96 × 128). During the rs-fMRI acquisition, participants were asked to look at a cross on the screen, to let their thoughts go, and to refrain from falling asleep.

### Definition of the DMN nodes from the resting-state data using independent component analysis (ICA)

Functional images were preprocessed, and group-level spatial ICA was conducted on the entire sample of participants. ICA was the chosen spatial analysis method for two reasons: (1) it represents a data-driven approach allowing the isolation of resting-state networks without any *a priori* hypothesis [71] and (2) it allows for the identification and removal of artifacts caused by motion from the fMRI signal [72].

Functional images were preprocessed using the same technique we applied in our previous study [32] using Statistical Parametric Mapping (SPM8, http://www.fil.ion.ucl.ac.uk/spm/). Briefly, functional images were realigned with respect to the mean image. The T1-weighted anatomical image of each individual was co-registered to the mean functional image and then segmented. The anatomical images and the functional realigned images were spatially normalized to the Montreal Neurological Institute (MNI) template, followed by spatial smoothing using an isotropic Gaussian smoothing kernel with a full width at half maximum (FWHM) of 6 mm. To avoid our results being biased by motion artifacts, we included only participants with movement less than 3 mm for translation and 3° for rotation. These criteria have widely been employed in normal and clinical populations [32, 48, 70, 73–79]. Mean translational and rotational movement, root mean squares (RMSs), and significance of the between-group difference for each direction is reported in Additional file 1: Table S2. No between-group significant differences were observed. However, the mean framewise displacement (FD) was significantly different between groups (mean FD controls = 0.086 ± 0.035, mean FD 22q11DS = 0.127 ± 0.068, *p* = 0.0005).

**Fig. 1:**
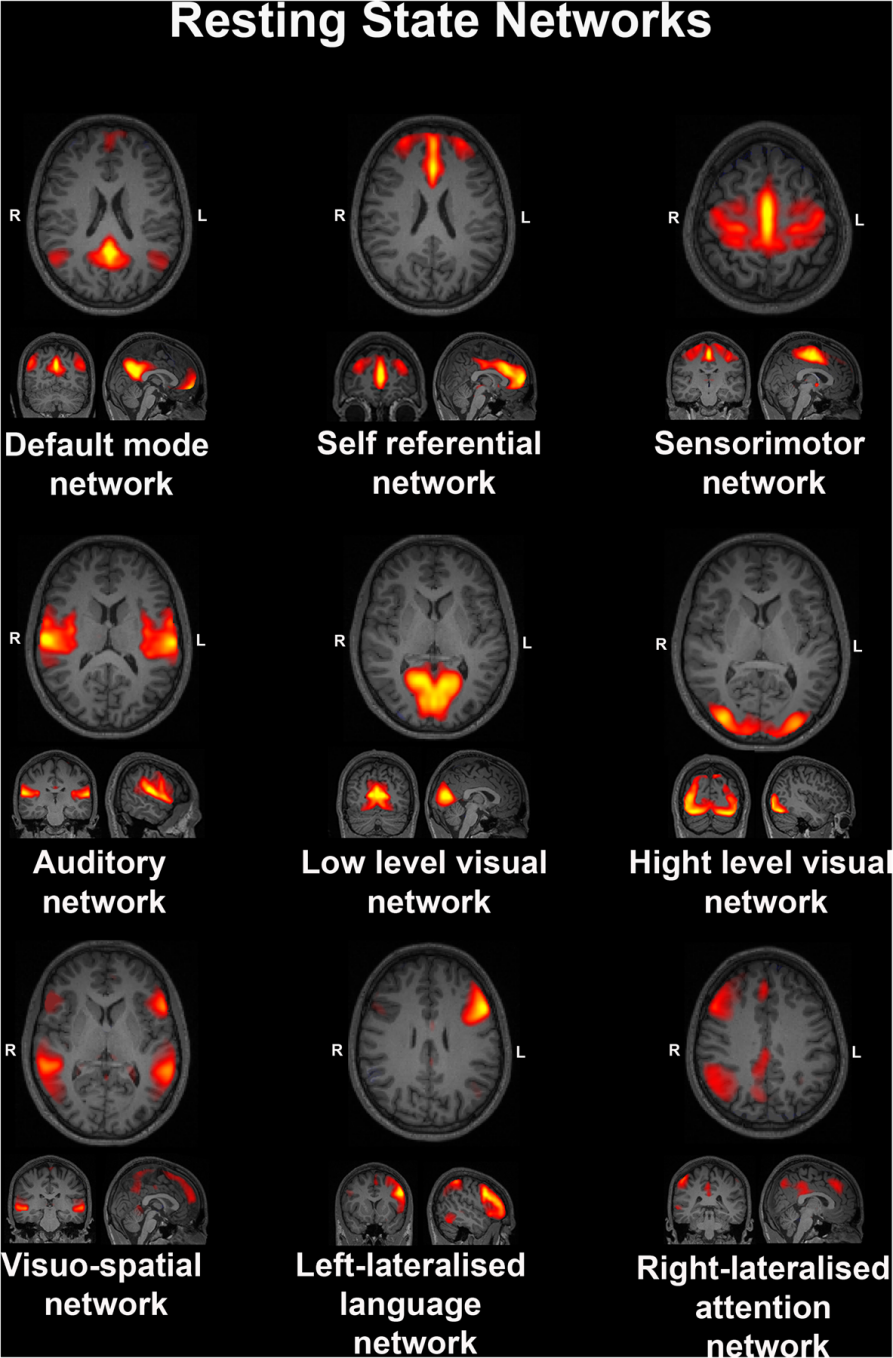
Nine resting-state networks (RSNs), which resulted from the independent component analysis (ICA)

Group-level spatial ICA was conducted on the entire sample of participants using the GIFT toolbox (http://mialab.mrn.org/software/gift/index.html). The number of components was fixed at *n* = 20 and, in accordance with our previous paper [32], 9 of the 20 components resulting from the ICA were visually identified as RSNs (Fig. 1). The remaining 11 components were considered artifacts due to motion or signal from the ventricles. Network identification was performed by visual inspection and confirmed by computing the correlation coefficient between the component and resting-state network templates (http://findlab.stanford.edu/functional_ROIs.html). Among the maps corresponding to the RSNs, only one was identified as the DMN and it comprised different clusters distributed along anterior and posterior medial brain regions and in two lateral regions corresponding to the IPL.

The map corresponding to the DMN was back reconstructed to obtain subject-specific DMN maps, converted into spatial Z-scores, thresholded at *z* = 2, and unwrapped in the subjects’ native space.

### Analysis of DMN connectivity: structural connectivity (DTI)

Data preprocessing and analysis were performed using the FSL Diffusion Toolbox (http://fsl.fmrib.ox.ac.uk/fsl/fslwiki/). The Brain Extraction Tool (BET, [80]) was employed for skull and non-brain tissue stripping. The effect of head motion and distortion of eddy currents was corrected using an affine alignment of all the weighted diffusion images onto the b0 image. The FMRIB’s Linear Image Registration Tool (FLIRT, [81, 82]) was used to register the T1-weighted images on the set of diffusion images.

The different clusters included in the DMN maps were separated to obtain independent images. Only the clusters with a dimension ≥400 voxels (corresponding to a volume of 325 mm^3^) were retained. Through visual inspection, each cluster was categorized as part of the anterior, posterior, or lateral components of the DMN. The position of each cluster was further confirmed by calculating the percentage of overlap between the cluster and four atlas-based (AAL atlas, [83]) structural regions of interest (ROIs) that were constructed in order to subdivide the whole brain into an anterior (frontal lobe and ACC), a posterior (occipital lobe, PCC, cuneus, precuneus), and two lateral regions (bilateral parietal lobes). In order to include the clusters corresponding to the main DMN regions, we only retained the clusters that overlapped for more than 50 % with one of the structural ROIs. Four masks corresponding to the mPFC/ACC, PCC/precuneus, and bilateral IPL were then binarized and used for fiber tracking.

Structural connectivity was estimated for each pair of regions (anterior and posterior DMN nodes and left and right IPL for a total of six connections) using the Probtrackx probabilistic tractography software from FSL [84]. The tractography algorithm drew 5000 streamline samples, with a step length of 0.5 mm and a curvature threshold of 0.2, from each voxel in the seed ROI to build up a connectivity distribution between the seed and the target ROI. This was repeated in the reverse direction (i.e., from target ROI to seed ROI), and the outputs of the two runs were averaged in order to obtain (1) the mean number of tracts connecting seed and target and (2) individual maps in which each voxel value indicated the probability of having fibers connecting the seed and the target region. Using the probabilistic maps, we computed a voxel-based connectivity measure, which is sensitive not only to the integrity and coherence of the white matter tracts but also to the tract geometry and length [85]. This mean connectivity index was calculated after thresholding the probabilistic maps to 25 % of their maximum value and calculating the mean of the thresholded maps. Additionally, mean FA along the tracts was calculated after masking the FA maps on the thresholded connectivity maps.

For the statistical analysis, we first assessed the normal distribution of the data corresponding to three structural connectivity measures (mean number of fibers, mean connectivity index, mean FA) using SPSS software (http://www-01.ibm.com/software/analytics/spss/). The mean number of fibers and the mean connectivity index were non-normally distributed, so we used two different approaches: (1) a non-parametric analysis of covariance [86] and (2) a logarithmic transformation of the data, in order to improve their normality, followed by a parametric analysis of covariance (ANCOVA). Since the mean FA values were normally distributed, we used an ANCOVA for analyses involving FA. Group comparisons were conducted using age, gender, and white matter volume as covariates. We decided not to use IQ as a covariate in the model for two reasons: (1) cognitive impairments are an integral part of the clinical profile in 22q11DS, so the effect of IQ reduction cannot easily be disentangled from other factors to explain the clinical and neural phenotype and (2) as expected from the first assumption, IQ significantly differs between patients and controls in our sample (*p* < 0.01), and it has been argued that it is not correct to include in the model a factor that significantly differs between groups [87].

In order to investigate the development of the DMN connectivity, we correlated the structural and functional connectivity measures with age in both 22q11DS and control groups. Given the wide age range of the participants included in the study, we subdivided the entire group of subjects into three different age bins and assessed the presence of between-group differences in structural connectivity measures using ANCOVA while covarying for age, gender, and white matter volume.

### Analysis of DMN connectivity: functional connectivity

To avoid spurious correlations due to non-neural signal or motion, functional scans were preprocessed adding additional steps using the DPARSF toolbox (http://rfmri.org/DPARSF). After removing the first five volumes, the functional images were realigned, segmented, and co-registered. In addition, the signal was linearly detrended and white matter, cerebro-spinal fluid signals, and movement were regressed out. The signal was then bandpass-filtered (0.01–0.1 Hz).

ROIs were constructed for each subject in native space. Spheres with an 8-mm radius were defined using the center of gravity coordinates of the four DMN clusters identified by ICA (anterior and posterior medial regions, bilateral IPL) as seed points. This methodology has been previously adopted [48] and provides a reliable measure of the region-averaged time series by increasing the signal-to-noise ratio. Functional connectivity between pairs of DMN nodes was measured using partial correlation. The result was a 4 × 4 matrix in which each element represented the connectivity strength between two regions controlled for the effect of the other regions. Fisher’s r-to-z transformation was applied to improve normality. Statistical analysis was conducted using ANCOVA covarying for age and gender.

## Results

### Group differences in structural connectivity

**Fig. 2:**
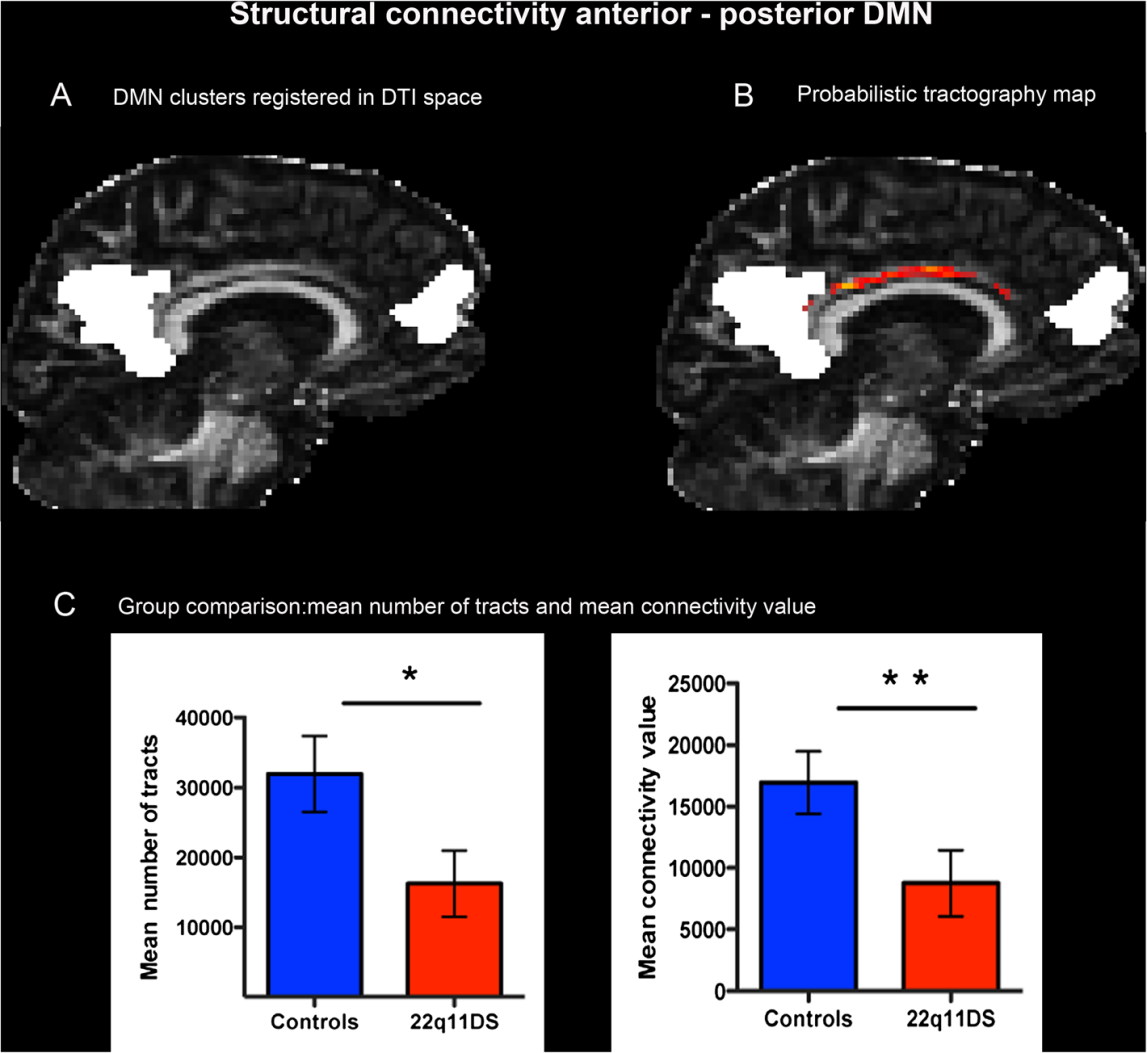
Structural connectivity between the anterior and the posterior node of the DMN (default mode network). DMN clusters were registered in DTI space (**a**) and used as regions of interest (ROIs) for the probabilistic tractography analysis (**b**). The connectivity map is represented in *red*/*yellow colors* and overlaid onto the fractional anisotropy (FA) map. The lighter the color, the greater the probability to have a connection. Structural connectivity measures (**c**) were significantly reduced in the 22q11DS population compared with controls (*p* < 0.05, indicated by *, *p* < 0.01, indicated by **)

Detailed descriptions of the structural connectivity measurements are displayed in Table 2. In the group of patients with 22q11DS, we observed a reduced number of tracts and mean connectivity index between the anterior and posterior medial regions of the DMN and between the anterior node of the DMN and the left IPL (Fig. 2). In order to assess if medication was affecting the results, we repeated the analysis after excluding the patients that were taking any psychotropic medication at the time of the visit. The results of the statistical analysis are reported in the Additional file 1: Table S3. Even with this smaller sample size, the results remained qualitatively the same. The mean FA values for both tracts did not show any significant reduction (*p* > 0.52) in the 22q11DS population.

**Table 2:** Statistical analysis of structural connectivity

	Controls	22q11DS	*F*_*(1,82)*_	*p*
Quade’s test				
Mean number of tracts				
Anterior-posterior DMN	31 957.84	16 266.24	5.742	0.019
Anterior DMN-left IPL	864.1	120.46	11.561	0.001
Mean connectivity value				
Anterior-posterior DMN	16 948.52	8 751.93	7.716	0.007
Anterior DMN-left IPL	282.81	43.932	11.413	0.001
ANCOVA on logarithmic transformed data				
Mean number of tracts				
Anterior-posterior DMN	4.25	3.82	8.661	0.004
Anterior DMN-left IPL	2.41	1.62	15.747	0
Mean connectivity value				
Anterior-posterior DMN	3.97	3.5	10.976	0.001
Anterior DMN-left IPL	1.9	1.21	15.039	0

**Fig. 3:**
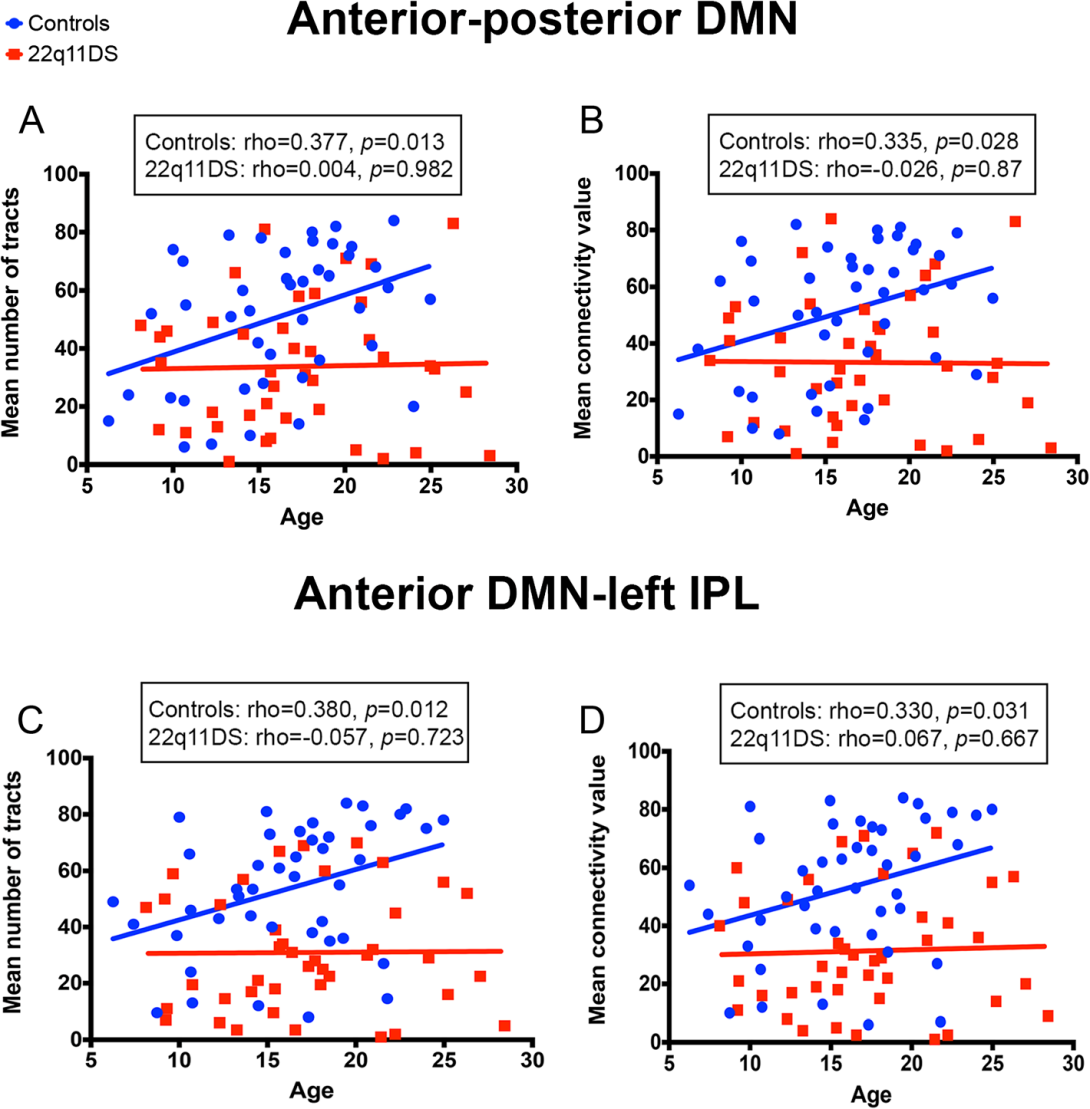
Correlation between structural connectivity and age. In the anterior-posterior (**a**, **b**) and anterior-left (**c**, **d**) default mode network (DMN) structural connectivity increases with age in control participants but not in patients with 22q11DS

**Fig. 4:**
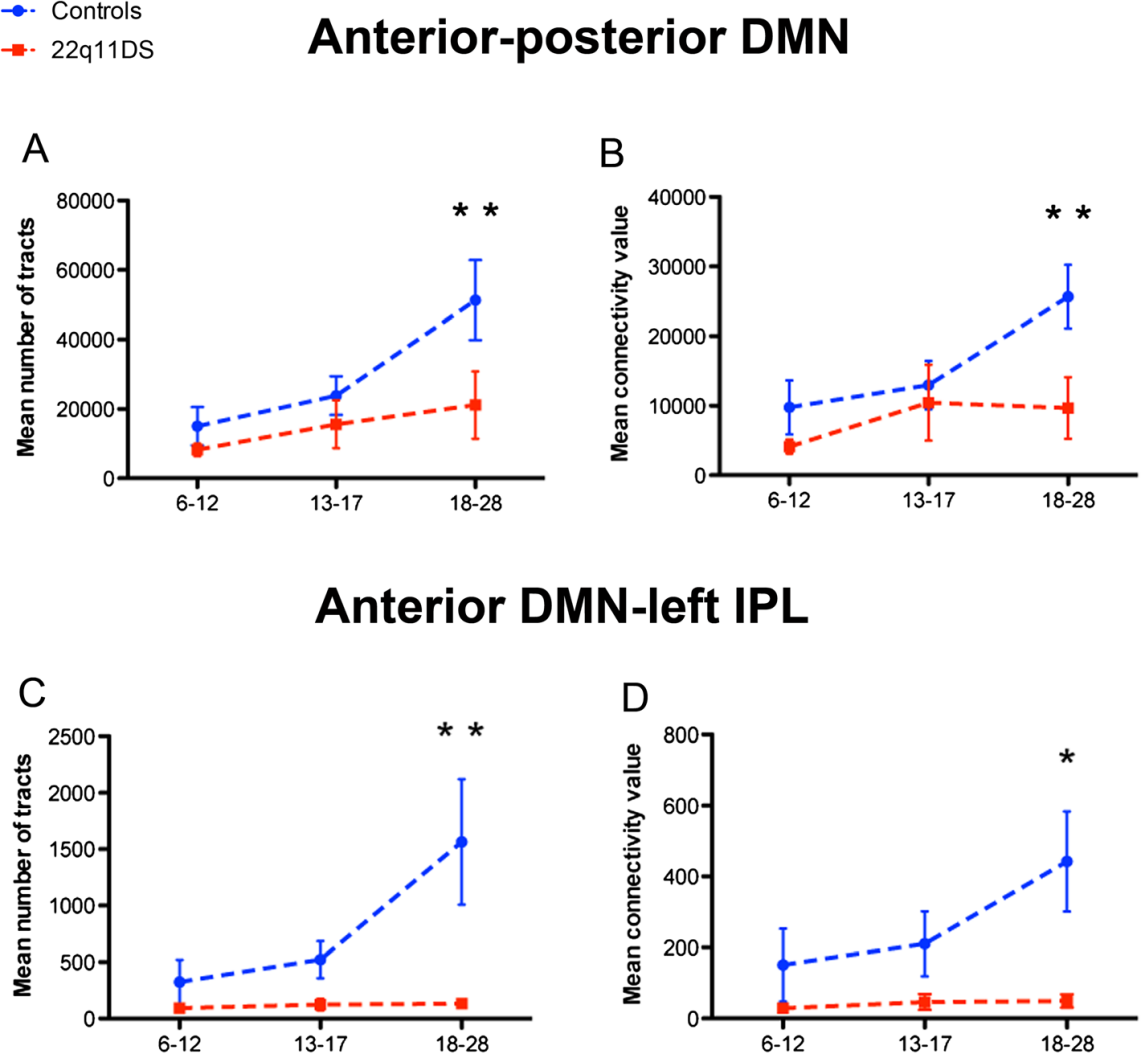
Structural connectivity in different age bins (6–12, 13–17, 18–28 years old). The mean number of tracts (**a**, **c**) and the mean connectivity values (**b**, **d**) in the anterior-posterior and anterior-left default mode network (DMN) connections were significantly reduced in the adult population (*p* < 0.05 indicated by *, *p* < 0.01, indicated by **)

For the connections that showed significant differences, namely between the anterior and posterior DMN and between the anterior DMN and the left IPL, we further assessed if the number of tracts and the mean connectivity index changed with age. The correlation analysis revealed that both measures were significantly correlated with age in the control group, but not in the group of patients with 22q11DS (Fig. 3). To further explore the effect of age, we subdivided the whole cohort of participants into three age bins: children (6–12 years old; 10 control participants, 9 patients), adolescents (13–17 years old; 17 control participants, 15 patients), and young adults (18–28 years old; 16 control participants, 17 patients). We observed that structural connectivity was preserved in children and adolescents affected by the syndrome whereas, in the group of adults, the mean number of tracts and the mean connectivity value were both significantly reduced (see Table 3 and Fig. 4), thereby suggesting an alteration in the maturation process of these white matter tracts.

**Table 3:** Statistical analysis of structural connectivity in the adult population

	Controls	22q11DS	*F*_*(1,31)*_	*p*
Quade’s test				
Mean number of tracts				
Anterior-posterior DMN	51 245.97	21 133.62	9.901	0.004
Anterior DMN-left IPL	1 565.47	131.47	8.913	0.005
Mean connectivity value				
Anterior-posterior DMN	25 664.69	9 698.67	12.992	0.001
Anterior DMN-left IPL	442.3	49.74	6.997	0.013
ANCOVA on logarithmic transformed data				
Mean number of tracts				
Anterior-posterior DMN	4.54	3.84	8.179	0.008
Anterior DMN-left IPL	2.69	1.69	10.941	0.003
Mean connectivity value				
Anterior-posterior DMN	4.27	3.51	11.044	0.002
Anterior DMN-left IPL	2.15	1.32	9.326	0.005

### Group differences in functional connectivity

**Fig. 5:**
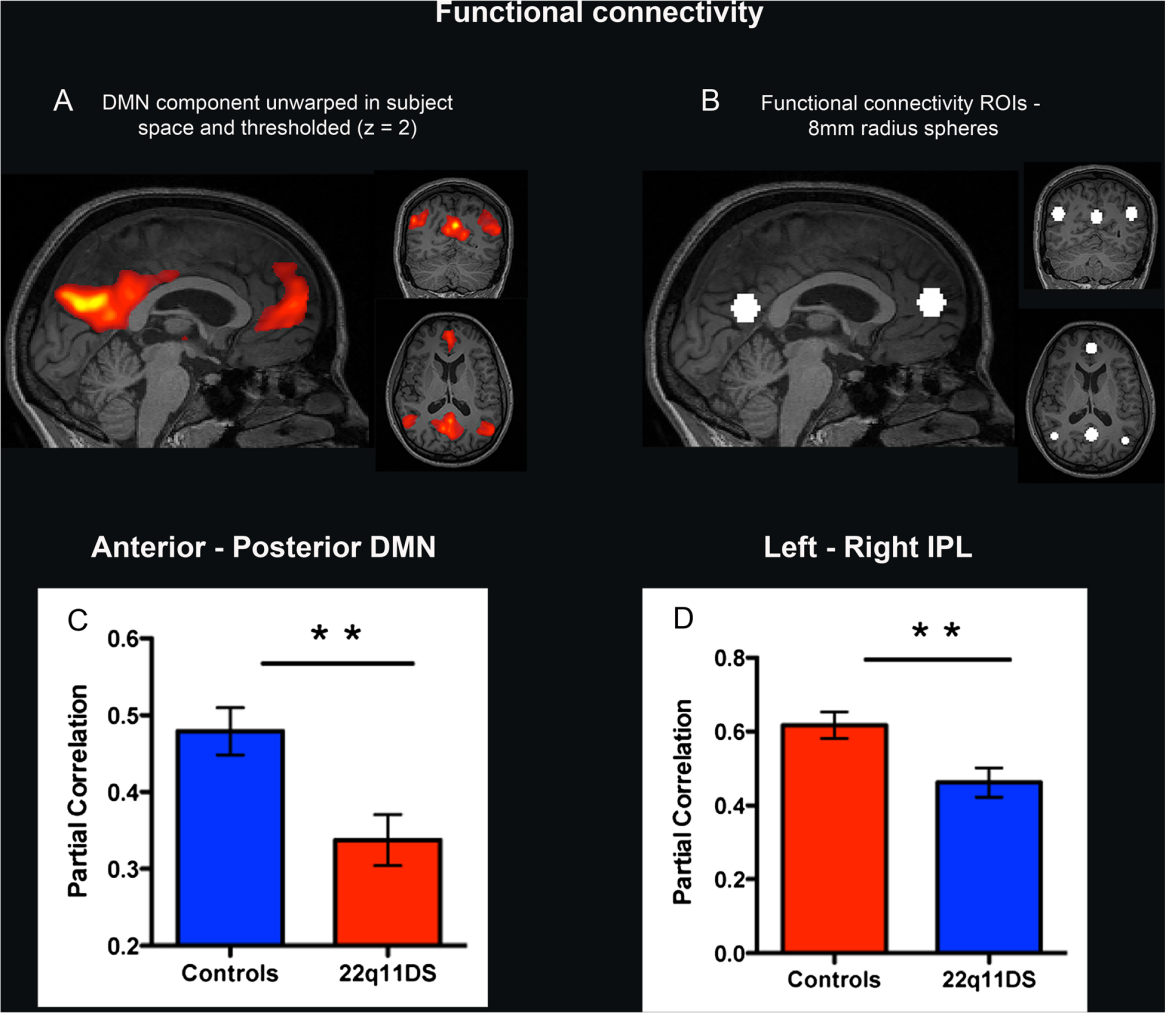
Functional connectivity analysis. The component corresponding to the default mode network (DMN) was isolated, unwrapped in subject space, and thresholded at *z* = 2 (**a**). Spheres of 8-mm radius were used as regions of interest (ROIs) for the functional connectivity analysis (**b**). Partial correlation revealed weaker functional connectivity between the anterior and the posterior DMN nodes (**c**) and between the left-right IPL (**d**) in the 22q11DS group compared to the control group (*p* < 0.01, indicated by **)

As for the structural connections, partial correlation revealed a significant reduction of functional connectivity between the anterior and the posterior medial regions of the DMN (*p* = 0.0025) and between the left and right IPL (*p* = 0.0055) in the group of patients with 22q11DS (Fig. 5). These results remained stable after covarying for the mean framewise displacement and for the Friston 24 motion parameters. There were no significant correlations between functional DMN connectivity and age (*p* > 0.52) in either of the two groups.

### Correlation between structural and functional DMN connectivity

We used the Spearman correlation coefficient to evaluate the presence of an association between structural and functional connectivity measures between the anterior and the posterior medial nodes of the DMN. There were no significant correlations in both groups (*p* > 0.366 in controls and *p* > 0.317 in 22q11DS). The analysis was then repeated using the three age subgroups but, also in this case, no evidence for a significant correlation between structural and functional DMN connectivity was found (*p* > 0.74 in children controls, *p >* 0.38 in children with 22q11DS, *p* > 0.65 in adolescent controls, *p* > 0.62 in adolescents with 22q11DS, *p* > 0.078 in adult controls, *p* > 0.56 in adults with 22q11DS).

### Correlation between DMN connectivity and prodromal psychotic symptoms

The strength of functional and structural DMN connections was not correlated with any of the five positive symptoms scales of the SIPS in our group of patients with 22q11DS (*p* > 0.24).

## Discussion

To the best of our knowledge, this is the first multimodal study investigating DMN structural and functional connectivity in 22q11DS. To date, studies on brain connectivity in patients with the syndrome were based on whole-brain approaches and adopted only one imaging modality, which was either rs-fMRI [32, 33, 70] or DTI [19–31]. Although not focused on the DMN, some of the abovementioned studies reported impaired connectivity within DMN regions [20, 22, 27, 28, 31]. Only one study specifically investigated the DMN functional connectivity in 22q11DS [33], confirming the presence of weaker connections between the anterior and posterior nodes. Using a data-driven approach, we reported a concomitant reduction of functional and structural connectivity between mPFC/ACC and PCC/precuneus in patients with 22q11DS.

Multimodal studies conducted in healthy subjects put forward an association between DMN functional and structural connectivity [48–52, 88], further reporting that this correlation became significant only in adulthood [48]. In our sample, we did not observe a direct correlation between the two connectivity measures, possibly due to the wide age range. Also, the relationship between structural and functional connectivity is complex, and it is therefore difficult to decipher whether alterations in the white matter tracts are responsible for the impairment in the functional connections or vice versa [89]. A previous study [33] reported the presence of stronger functional connectivity between DMN and non-DMN brain regions in 22q11DS, suggesting that impairments in the white matter tracts connecting DMN regions can lead to plastic compensatory adaptations resulting in the development of alternative functional pathways [90]. Studies conducted in animal models would be useful in further clarifying this relationship.

Our findings indicate a disruption in the DMN connectivity in 22q11DS and are consistent with previous evidence collected in patients affected by other neurodevelopmental disorders such as autism spectrum [44, 45] and attention deficit hyperactivity [47, 56] disorders, fragile X syndrome [55], and schizophrenia [38–43, 46, 53]. In particular, two studies simultaneously investigated functional and structural DMN connectivity in schizophrenic patients [53, 54]. While Chamchong et al. [53] showed a concomitant reduction of both connectivity measures, Skudlarski and colleagues [54] found a lower coherence between the DMN functional and structural connections in those with schizophrenia, thereby suggesting that schizophrenic patients develop new patterns of functional connectivity not related to underlying structural connections.

In both studies, functional and structural DMN disconnectivity were associated with the severity of psychotic symptoms [53, 54]. In our group of patients, however, we did not find evidence for a correlation between the DMN structural and functional connectivity impairments and the manifestation of early symptoms of psychosis. In a previous study with a partially overlapping cohort of adolescent patients [32], we observed that increased activity in the left superior frontal gyrus (lSFG) was associated with severity of early psychotic symptoms. However, our present analysis differs from the previous by Debbané and colleagues for two main aspects. First, although the lSFG is considered as part of the DMN, our data-driven approach did not include this region in the anterior node of the DMN. Second, unlike our previous study, we looked at the connectivity between pairs of DMN nodes and not at the activation of a single DMN region. One possible explanation for the lack of correlation between DMN connectivity and SIPS scores in the present study is that we might not have sufficient power. Indeed, as reported in Additional file 1: Table S1, the mean symptom intensity in our group was relatively low. Moreover, the small number of subjects having major to severe psychotic symptoms (three affected by a psychotic disorder and five with an UHR) prevented the possibility of comparing the DMN connectivity between patients with and without such symptoms*.* Given the dynamic nature of neurodevelopmental trajectories in psychotic spectrum disorders, future longitudinal studies may further observe different connectivity patterns at different stages of progression of the disorder.

Moreover, we observed that structural connectivity between the anterior and the posterior DMN nodes did not typically develop with age in 22q11DS, supporting the claim that maturation of brain connectivity is impaired in patients affected by neurodevelopmental diseases [91]. In line with previous findings in healthy subjects [48, 61, 92], we report an age-related increase of the DMN structural connectivity in our control group. However, we did not observe a similar trend of increased connectivity with age in 22q11DS (Fig. 3). Consequently, structural connectivity did not differ between patients and controls in the groups of children and adolescents; however, reduced structural connectivity was observed in adults affected by 22q11DS (Fig. 4). Such findings largely corroborate previous observations of deviant developmental trajectories in 22q11DS, using different imaging modalities [58–60, 93], including our previous finding of a lack of age-related increase in FA in patients with 22q11DS [31]. Also, a study using ^1^H spectroscopy [60] reported an absence of age-associated decrease in the NAA levels of patients with 22q11DS, indicating alterations in cortical development. Anatomical studies also provided evidence of abnormal neurodevelopmental trajectories in 22q11DS, such as volumetric impairments in gray matter maturation during the transition into late adolescence-young adulthood [58, 59] and greater cortical loss during adolescence [57]. The currently observed absence of typical increase in structural connectivity with age adds an additional piece to the complex pattern of cerebral maturation associated with 22q11DS during development.

## Conclusions

This study reported original evidence for a concomitant reduction in structural and functional connectivity between core medial nodes of the DMN in 22q11DS. Additionally, we found that structural DMN connections did not typically develop with age in patients with 22q11DS, confirming the presence of altered developmental trajectories already reported in this group of patients.

However, there were several limitations to the current study. Firstly, we decided to employ a probabilistic tractography algorithm [84] to partially reduce the problem of crossing fibers. However, as discussed in [31], tractography studies bear some limitations. Another issue concerns head motion. Indeed, with exclusion criteria of 3-mm translation and 3° rotation, we excluded 55 subjects. Even after excluding the subjects that moved most, the mean framewise displacement remained significantly greater in the group of patients than in the control participants. Possible factors that can explain motion in our sample are the length of our scanner protocols (50–70 min including a high-resolution T1-weighted MRI, a diffusion acquisition, resting-state fMRI, and functional task), the young age of the participants, or the presence of cognitive deficits and psychotic symptoms. Therefore, using more stringent selection criteria may introduce a bias in our analysis, resulting in the inclusion of the oldest patients with less comorbidity. In order to account for motion artifacts, we further performed several steps (no between-group difference in mean translation and rotation and regression of motion parameters), but we cannot totally exclude the effect of residual motion on the results*.* A third limitation is that the mean IQ values significantly differ between patient and control groups. Although the connectivity measures did not correlate with IQ, thereby suggesting that the group differences cannot be attributed to differences in IQ values, only the recruitment of IQ-matched control participants could confirm this. Finally, although our findings indicate impaired maturation of the white matter tracts connecting the DMN in 22q11DS, longitudinal studies are necessary to confirm the developmental trajectories of white matter maturation.

## Additional file

Additional file 1:
**Supplementary tables.** Table S1. Mean SIPS scores for positive and negative symptoms subscales. Table S2. Functional data motion parameters. Table S3. Statistical analysis of structural connectivity after excluding the subjects receiving medications at the time of the visit.
