# Supplementary material for: Structural and functional connectivity in the default mode network in 22q11.2 deletion syndrome
Source: J Neurodev Disord. 2015 Aug 1;7(1):23. doi: 10.1186/s11689-015-9120-y (PMC4522079; doi:10.1186/s11689-015-9120-y)
Supplement: Additional file 1: — Supplementary tables. Table S1. Mean SIPS scores for positive and negative symptoms subscales. Table S2. Functional data motion parameters. Table S3. Statistical analysis of structural connectivity after excluding the subjects receiving medications at the time of the visit. [file 11689_2015_9120_MOESM1_ESM.docx]

**Table S1. Mean SIPS scores for positive and negative symptoms subscales**

| **Positive symptoms subscales** | **Mean ± SD** |
| --- | --- |
| P1 (unusual thoughts) | 1.41 ± 1.67 |
| P2 (suspiciousness) | 1.65 ± 1.50 |
| P3 (grandiose ideas) | 0.15 ± 0.36 |
| P4 (hallucinations) | 1.42 ± 1.75 |
| P5 (disorganized communication) | 0.54 ± 0.97 |
| **Negative symptoms subscales** |  |
| N1 (social anhedonia) | 2.33 ± 1.43 |
| N2 (avolition) | 2 ± 1.12 |
| N3 (expression of emotion) | 2.36 ± 1.21 |
| N4 (experience of emotion and self) | 0.76 ± 1.09 |
| N5 (ideational richness) | 3.61 ± 1.39 |
| N6 (occupational functioning) | 2.42 ± 1.20 |

**Table S2. Functional data motion parameters.**

|  |  | Controls | 22q11DS | *P*-value |
| --- | --- | --- | --- | --- |
| Mean translation | x | 0.02761±0.1303 | 0.04122±0.1918 | 0.0806 |
|  | y | 0.07645±0.1930 | 0.00104±0.2162 | 0.5036 |
|  | z | 0.16477±0.3087 | 0.10216±0.4386 | 0.1793 |
| Mean rotation | x(pitch) | 0.00234±0.0053 | 0.00197±0.0068 | 0.1610 |
|  | y(roll) | 0.00074±0.0036 | 0.00111±0.0045 | 0.1528 |
|  | z(yaw) | 0±0.0033 | 0.00117±0.0061 | 0.1745 |
| RMS translation | x | 0.09928±0.1138 | 0.15324±0.1594 | 0.0768 |
|  | y | 0.20154±0.1391 | 0.17785±0.1782 | 0.4977 |
|  | z | 0.3259±0.2805 | 0.40386±0.3173 | 0.2358 |
| RMS rotation | x(pitch) | 0.00589±0.0037 | 0.00725±0.0048 | 0.1460 |
|  | y(roll) | 0.0031±0.0029 | 0.00411±0.0033 | 0.1369 |
|  | z(yaw) | 0.00288±0.0026 | 0.00432±0.0057 | 0.1381 |

**Table S3. Statistical analysis of structural connectivity after excluding the subjects receiving medications at the time of the visit.**

| **Quade’s test** | | | | |
| --- | --- | --- | --- | --- |
|  |  |  |  |  |
|  | Controls (n=43) | 22q11DS (n=25) | *F _(1,68)_* | *p* |
| **MEAN NUMBER OF TRACTS** | | |  |  |
| Anterior - Posterior DMN | 31 957.84 | 16 485.30 | 3.03 | 0.086 |
| Anterior DMN - Left IPL | 864.1 | 142.02 | 3.95 | 0.051 |
| **MEAN CONNECTIVITY VALUE** | | |  |  |
| Anterior - Posterior DMN | 16 948.52 | 7 632.49 | 5.01 | 0.029 |
| Anterior DMN - Left IPL | 282.81 | 56.72 | 3.98 | 0.050 |
| **ANCOVA on logarithmic transformed data** | | | | |
|  |  |  |  |  |
|  | Controls (n=43) | 22q11DS (n=25) | *F _(1,68)_* | *p* |
| **MEAN NUMBER OF TRACTS** | | |  |  |
| Anterior - Posterior DMN | 4.25 | 3.91 | 4.34 | 0.041 |
| Anterior DMN - Left IPL | 2.41 | 1.84 | 4.52 | 0.037 |
| **MEAN CONNECTIVITY VALUE** | | |  |  |
| Anterior - Posterior DMN | 3.97 | 3.56 | 7.09 | 0.010 |
| Anterior DMN - Left IPL | 1.9 | 1.39 | 4.04 | 0.049 |
